# Editorial: Roots—The Hidden Provider

**DOI:** 10.3389/fpls.2017.01021

**Published:** 2017-06-13

**Authors:** Janin Riedelsberger, Michael R. Blatt

**Affiliations:** ^1^Centro de Bioinformática y Simulación Molecular, Universidad de TalcaTalca, Chile; ^2^Laboratory of Plant Physiology and Biophysics, University of GlasgowGlasgow, United Kingdom

**Keywords:** roots, ion transport, root system architecture (RSA), modeling biological systems

Most plant roots are found hidden underground. They can form immense root systems with lengths of several kilometers featuring an architecture with up to millions of branch roots. Their versatile functions range from anchoring plants in soil via storing photosynthetic products to the vital uptake of water and nutrients. Plants build the basis of a food chain that ends with more than seven billion people who have to be fed every day. Therefore, it is vital to secure and enhance crop production, especially in terms of prevailing agricultural threats and climate change. The challenge must be to optimize plant's nutrient acquisition and stress tolerance. Understanding plant nutrition, homeostasis, and stress responses is a first step to directed and efficient manipulations and adjustments to counterbalance stress and deficiency symptoms. This knowledge must be used to optimize the use of fertilizer and water to achieve well-balanced nutrition acquisition as well as the use of pesticides to treat biotic stress. Such optimization is desirable as the constant use of fertilizer, irrigation, and pesticides is associated with negative long-term effects. Knowledge about nutrient uptake and signaling as well as behavior in stress situations will be valuable if we are to design adequate treatments to prepare plants for environmental challenges.

## Nutrient uptake and signaling

Nutrient uptake is the first step in the production of plant mass. It starts in the roots, which represent the main entrance for macro- and micronutrients that are crucial for elementary cell processes. Deficiency or excess nutrient uptake can result in severe damage to the point of yield loss or even plant death. Therefore, a balanced nutrient supply and its uptake are essential for optimal plant growth and crop production. Nutrient distribution in the soil is heterogeneous in space and time and affected by many factors, including binding and sequestration, groundwater level, pH, and salinity.

The three primary plant macronutrients are potassium, nitrate, and phosphorus. Over the course of evolution, transport systems with differing affinities for nutrients evolved to cope with variations in nutrient availability. Nieves-Cordones et al. and Noguero and Lacombe review high- and low-affinity uptake systems for potassium and nitrate, respectively. The uptake of phosphorus, which serves as well as nitrogen as constituent of macromolecules is reviewed by Gu et al. (2016).

Nitrate also acts as signaling molecule influencing lateral root primordia and root branching (Noguero and Lacombe). A more extensive signaling system is built upon calcium. The specificity of calcium signaling is determined by patterns of cytosolic calcium concentrations taking into account at least the amplitude and duration of calcium increases. Cytosolic free calcium concentration acts as a secondary messenger in the signaling of abiotic stress including nutrient deficiency. The comprehensive review of Wilkins and colleagues summarizes the state of the art of calcium's broad modes of operation. Besides, calcium transport systems, that are still an active field of investigation, are reviewed (Wilkins et al.).

Micronutrients often occur abundantly in solid earth but mainly in an insoluble form that is not suitable for uptake, meaning that plants may suffer nutrient deficiency despite their presence in the soil. One example is iron. Plants exhibit a number of special uptake strategies for iron. Poaceae use a chelation strategy to uptake iron by secreting phytosiderophores or precursors of phytosiderophores into the rhizosphere. Then, the chelated iron(III)-phytosiderophore complex is taken up into the plant by special transport systems. Non-Poaceae use an acidification-reduction strategy, which acidifies the rhizosphere to reduce iron(III) to iron(II), which can be taken up by plant roots. Phenolic compounds like coumarins, especially catechol coumarins, have been reported to play a role in iron recruitment showing iron(III) reducing and iron(II) chelating activity (Sisó-Terraza et al.).

Generally, acidic soils stimulate the uptake of micronutrients like iron. However, low pH also increases the solubility of toxic metals like aluminum, which directly affects the root system architecture (Rao et al., 2016). To control toxic metals plants developed quite sophisticated defense mechanisms. For example, plants chelate toxic aluminum ions with malate and export the aluminum-malate complex (Sharma et al.). ALMT channels have been identified to transport these malate-aluminum complexes. Latest research showed that the ALMT anion channel family has a far more versatile functional spectrum than initially thought. Sharma and colleagues give an extensive overview on ALMT's variable physiological and structural aspects including their input in mineral nutrition, ion homeostasis, and guard cell function among others.

## Plant root—microbe interactions

The most important plant-microbe interaction is the mycorrhizal symbiosis between plants and fungi. Plants benefit from enhanced nutrient uptake and stress resistance, while the fungi are provided with photosynthetical products. Schott and colleagues described a minimal network of transporters that is sufficient to describe realistically the bidirectional nutrient trade system (Schott et al.).

Another important plant symbiotic relationship is that between legumes and rhizobia, a type of nitrogen-fixing bacteria. Here, the plant benefits from the biological nitrogen fixation that is carried out by the rhizobial symbiont, while bacteria receive photosynthetically fixed carbon. Establishing such interactions between plant root hairs and microorganisms is initiated by chemical communication. Bacterial symbionts respond to plant signaling compounds with nod factor secretion that induces broad changes in the plant root transport machinery (Damiani et al.). Using high throughput RNA sequencing Damiani and colleagues identified a set of transport systems that are likely to be involved in early nodulation (Nod) factor signaling suggesting substantial rearrangements in the nutrient transport machinery after Nod factor perception.

Root exudates may also contain substances that function as allelochemicals for plant's defense. Infestation with pathogenic microorganisms can lead to substantial yield losses. It is thus essential to understand how plants are able to resist pathogen invasion if we are to enhance these defense mechanisms. A common signal on the part of bacteria is the bacterial protein flagellin flg22 that is recognized by the plant receptor kinase FLS2 and also induces ALMT transporter expression (Chen et al.; Sharma et al.).

## Root system architecture responses to stress

Generally, plants are exposed to several stresses simultaneously. Although much attention is drawn to the characteristics of the shoot, effects on roots are often ignored. Though, root plasticity is highly flexible and its development is guided by environmental conditions like nutrient availability. Koevoets et al. address this gap in focus and summarize comprehensively the effects of nutrient deficiencies and other abiotic stresses on the root system architecture plasticity. In addition, the authors discuss challenges that have to be resolved to implement root system architecture evaluation in crop selection.

One of the major agricultural challenges is the increasing salinization of agricultural land. The majority of crop plants are salt-sensitive, which is why articles of this research topic concentrate on glycophytes. Nieves-Cordones and colleagues reviewed present knowledge on sodium uptake systems in plant roots and their regulations (Nieves-Cordones et al.). Sodium may be beneficial for plants, especially under potassium shortage, but it is not an essential nutrient. At high concentrations it causes stress that has far-reaching impact. Annunziata et al. and Jiang et al. present examples on how salt stress affects cytosolic metabolites and root growth.

## Root-shoot communication

For centuries the increase in crop yield raise and quality has been achieved by transferring the scion of one plant to the rootstock of another plant. This process, known as grafting, may lead to increased nutrient uptake and stress tolerance among other benefits. Despite long and still actively utilized practice of grafting, physiological, and molecular bases are not fully understood yet. Nawaz et al. summarizes grating effects on ion uptake and accumulation. The authors discuss the enhanced expression of transport-related genes and changes in hormonal levels that influence the architecture of root systems.

The root structure not only responds to external stimuli, but also to partitioning patterns of photoassimilates in roots. Mudgil et al. discuss a possible signaling mechanism of photosynthetically fixed sugars that affect root system architecture. It is vital to understand root-shoot communication since seed production, the origin of the coming generation, depends on optimal grain filling that in part is achieved by root carbon sources especially under dry conditions (Zhang et al.).

## Modeling of nutrient transport

Uptake and transport pathways of nutrients are well-organized for every mineral. These pathways intersect and result in complex networks that can be challenging to access experimentally. Modeling of transport processes, nutrient exchange and uptake processes have been subject to long-term investigations, which aim to predict and describe experimental data. Le Deunff and colleagues describe the advantage of the flow-force modeling approach over the enzyme-substrate approach by reviewing developments and advances carried out on modeling of nutrient uptake (Le Deunff et al.). Water and nutrient uptake into roots is non-uniform in space. Foster and Miklavcic illustrate root zone effects on passive water and nutrient uptake. Modeling approaches are advancing and more and more capable of reflecting biological realities. Schott and colleagues successfully reconstructed nutrient exchange processes between mycorrhizal symbionts and plant root cells. Their model furthermore permitted them to hypothesize about the potential mechanism behind symbiont-plant interactions (Schott et al.).

Modeling approaches go beyond the description of nutrient uptake and transport. Efforts have been made to simulate effects on whole root system architectures in response to nutrient availability (Postma et al., 2014a,b).

## Outlook

For future investigations it is crucial to combine studies of entire root systems and plants with modeling approaches. This ensures the assessment of systemic effects that result from changes in single traits. Advances in this field should be integrated into current experimental pipelines to refine and accelerate research. Further advances in the understanding of nutrient homeostasis are needed to enhance nutrient use efficiency and optimize the use of fertilizer as well as to improve and maintain soil quality and protect the environment from inefficient water and fertilizer use.

## Author contributions

JR wrote the first draft of the manuscript. JR and MB revised and improved the manuscript.

### Conflict of interest statement

The authors declare that the research was conducted in the absence of any commercial or financial relationships that could be construed as a potential conflict of interest. The reviewer KS and handling Editor declared their shared affiliation, and the handling Editor states that the process nevertheless met the standards of a fair and objective review.
